# Lymphadenovarix in the axilla – an unusual presentation of filariasis

**DOI:** 10.1186/1475-2883-5-9

**Published:** 2006-07-30

**Authors:** Adhish Basu, Sarath Chandra Sistla, Surendra Kumar Verma, S Jagdish

**Affiliations:** 1 Department of Surgery, Jawaharlal Institute of Postgraduate Medical Education and Research, Pondicherry, India; 2 Department of Pathology, Jawaharlal Institute of Postgraduate Medical Education and Research, Pondicherry, India

## Abstract

Clinical manifestations of lymphatic filariasis depend on the area of lymphatic involvement and the duration of infection. A 21 year old man, resident in a filariasis endemic region, presented with multiple matted lymph nodes with cystic areas forming a large mass in his left axilla. An ultrasound scan of the axilla using a 7.5 MHz transducer revealed grossly dilated lymphatics but no filarial dance sign. Fine needle (21 G) aspiration cytology (FNAC) from the dilated lymphatics and solid areas in the lymph node mass revealed multiple microfilariae in a background of reactive lymphoid cells. Peripheral blood smears revealed microfilaremia with significant eosinophilia. Diagnosis of left axillary *Bancroftian lymphadenovarix *was made. On the administration of oral diethylcarbamazine, the diameter of the lymphatic vessels in the lymphadenovarix reduced considerably in size and microfilaremia disappeared. We report this case because axillary lymphadenovarix is a rare presentation of filariasis. This case is also unique since microfilariae were demonstrated in the fluid aspirated from the dilated lymphatics of the lymphadenovarix in the absence of live adult worms.

## Introduction

Lymphatic filariasis is a major health problem in India especially in its southern provinces. A majority of the infected individuals in filariasis endemic communities are asymptomatic. Adult worms in the lymphatics cause progressive lymphatic vascular dilation and dysfunction [1]. Progressive lymphatic dysfunction usually presents as lower limb lymphoedema, hydrocele, chyluria or rarely groin lymphadenovarix [2]. Axillary lymphadenovarix is an extremely uncommon presentation of filariasis even in endemic communities. We thus present a case of filarial lymphadenovarix of the axilla which was successfully treated with diethylcarbamazine.

## Case report

**Figure 1:**
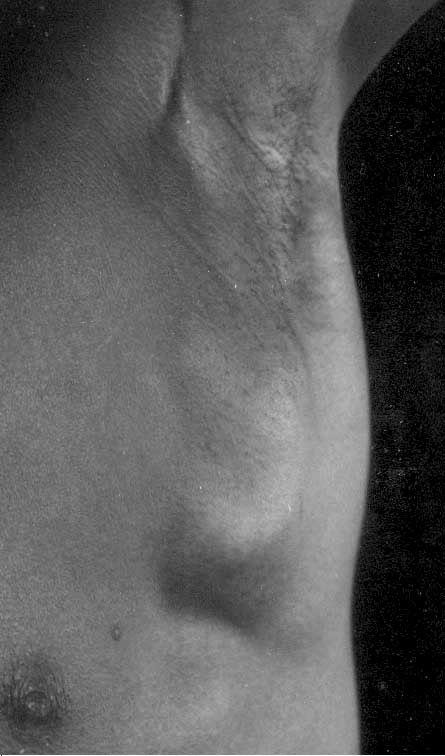
Left axillary mass at presentation.

A 21 year old presented in July 2004 complaining of a large painless lump in the left arm pit which he reported as a problem of six months duration. He was a manual laborer working from Tamil Nadu, India. He had no history of episodic fever, cough, weight loss, swelling of the left upper extremity or any lumps elsewhere in the body. Clinical examination revealed a mobile and non-tender mass of matted lymph nodes measuring 5 cm × 5 cm with heterogeneous (soft to firm) consistency with some cystic areas, in the central part of the left axilla (Figure 1). No other lymph node groups were significantly enlarged. External genitalia were normal. General examination revealed no other abnormalities. A clinical diagnosis of tubercular axillary lymphadenitis was entertained.

A chest x-ray was unremarkable. There was no evidence of leucocytosis, eosinophilia or parasites in his venous blood, sampled during the day. Routine and microscopic examination of the urine was within normal limits. Fine needle (21 G) aspiration cytology from the axillary mass revealed a scanty aspirate, smears from which showed features suggestive of reactive lymphoid hyperplasia.

**Figure 2:**
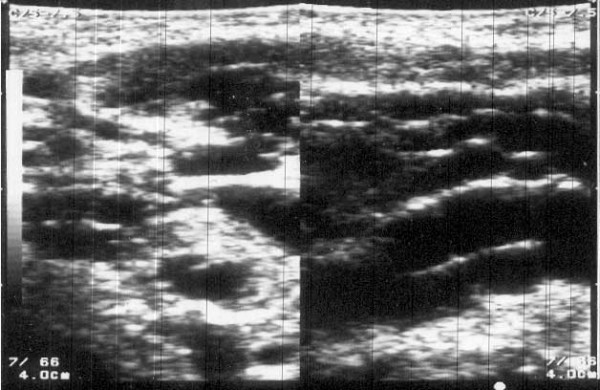
Ultrasound scan of axilla at presentation showing grossly distended lymphatics (in longitudinal and transverse axis).

**Figure 3:**
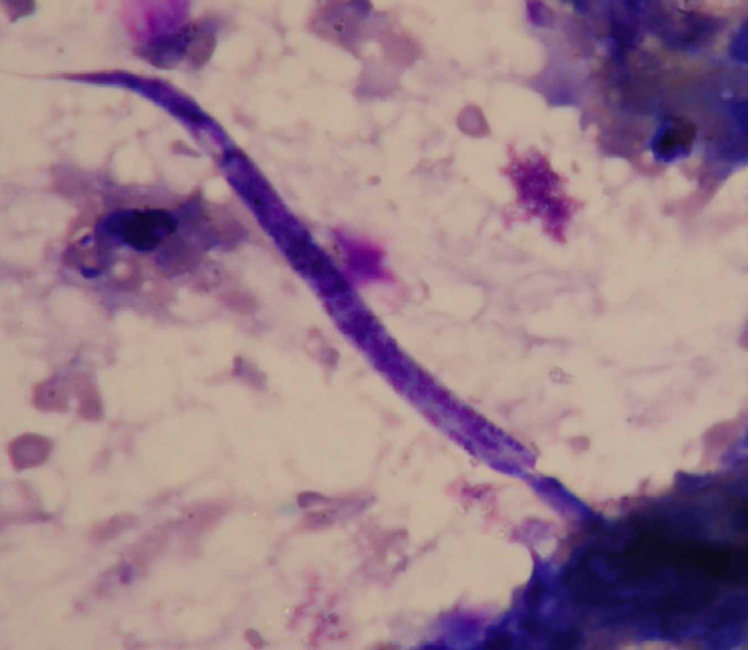
FNAC showing microfilariae of Wuchereria bancrofti in a background of reactive lymphoid cells.

An ultrasound scan of the left axilla in real-time B-mode and M-mode was carried out by an experienced sonologist in the presence of one of the authors (AB) with a Toshiba (Justvision 400) ultrasound machine (Toshiba Medical System Corporation, Togichi, Japan) and a 7.5 MHz linear phased array transducer. The scan revealed a lobulated heterogeneous soft tissue mass of size 5.5 cm × 4.5 cm × 5.0 cm. The mass consisted of solid hypoechoic areas (each 2 cm × 1 cm) with multiple dilated tortuous anechoic channels (suggestive of grossly dilated lymphatics) of varying diameter (7 mm to 10 mm). There was no evidence of calcification or the typical filarial dance sign of *Wuchereria bancrofti *inside the dilated channels (Figure 2). A sonographic diagnosis of the left axillary lymphadenopathy with lymphangiectasia was made. Fine needle aspiration cytology from the dilated lymphatics and the solid areas was then performed and microscopic examination of which revealed multiple microfilariae of *Wuchereria bancrofti *in a background of reactive lymphoid cells (Figure 3). A scrotal ultrasound scan was not done as the external genitalia were normal.

**Figure 4:**
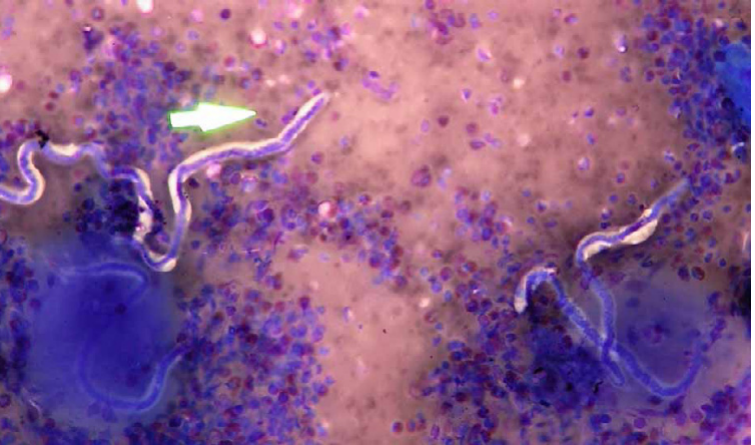
Peripheral blood smear showing microfilaremia and eosinophilia.

A nocturnal venous blood smear examination (three weeks after presentation) showed eosinophilia but failed to show any microfilaria. The absolute eosinophil count was 2520/mm^3^. A thick venous blood smear examination taken one hour after the administration of 100 mg oral diethylcarbamazine (in the daytime) (DEC provocation test) revealed numerous typical microfilariae of *Wuchereria bancrofti *(Figure 4). The microfilarial density however, was not determined.

**Figure 5:**
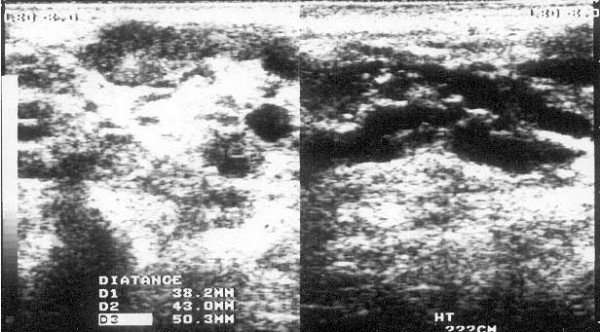
Ultrasound scan of axilla after therapy showing collapsed lymphatics (in longitudinal and transverse axis).

The patient then received oral diethylcarbamazine (100 mg q8 h) for 3 weeks. In October 2004 the size of the axillary mass and diameter of the lymphatic vessels had reduced in size considerably. This was confirmed by a repeat ultrasound examination of the left axilla (performed by the same sonologist using the same ultrasound machine), which revealed a heterogeneous soft tissue mass of 3.0 cm × 3.0 cm × 4.5 cm with dilated tortuous lymphatic channels, diameter less than 5 mm (Figure 5). The patient had no other symptoms or signs attributable to filariasis. No scrotal nodules developed during the period of treatment. Repeated microscopic examinations of thick smears of the patient's venous blood taken at night yielded no microfilaria. Membrane filtration concentration technique for detection of microfilaria was not used.

## Discussion

Lymphatic filariasis is a major health problem in India with most infections caused by *Wuchereria bancrofti*. The presence of adult worms of *Wuchereria bancrofti *in the infected individuals is confirmed by detecting microfilariae or filarial antigens in the patient's blood [3]. Ultrasound scans (B-mode and M-mode) with or without colour Doppler or pulse wave Doppler have been used to detect living adult *W. bancrofti *(filarial dance sign) in dilated intrascrotal juxtatesticular lymphatics (worm nests) of approximately 80% of microfilaremic but asymptomatic men residing in endemic areas [4]. In individuals from endemic regions, with no sonologically detectable worms or microfilaremia, the presence of dilated lymphatics in the scrotum has been shown to be due to occult adult worms in those lymphatics [1]. In these individuals treatment with diethylcarbamazine has led to the formation of scrotal nodules, histological examination revealing dead adult worms [3]. Microscopic examination of fine needle aspirates from dilated lymphatics harboring adult worms in symptomatic patients has also revealed microfilariae [5]. Similarly, microscopical examination of lymph node biopsies revealed microfilaria with an adult worm in the same lymphatic vessel in one case [6]. However, to the best of our knowledge there are no reports of fine needle aspirates from dilated lymphatics without detectable adult worms revealing microfilariae on microscopy.

The diagnosis of a filarial infection can also been made by detecting microfilariae on microscopic examination of fine needle aspirates from lymph nodes [7,8]. Fine needle aspiration cytology from breast mass, thyroid mass, hydrocoele fluid, pericardial fluid, pleural fluid, ascitic fluid, and cytology of cervicovaginal smears, bronchial aspirates, urine, nipple secretion, bone marrow and joint fluid aspirates have also been reported to yield microfilariae [9,10]. Moreover, in these patients the peripheral smears rarely revealed microfilaremia or eosinophilia [9,10].

Our case appears unique for a number of reasons. Firstly, filarial lymphangiectasia presenting as a visible lump (lymphadenovarix) in the axilla is rare. Secondly, the ultrasound scan of the lump did not identify any adult worm(s) in the axilla in spite of significant lymphangiectasia. The absence of worms in the lymphatics on the ultrasound does not, however, rule out their presence in the deeper lymphatics of the axilla. Interestingly, the filarial dance sign has not been detected in the axillary lymphatics of asymptomatic amicrofilaremic individuals residing in a filariasis endemic region of South India [11]. Thirdly, ultrasound guided aspiration from the dilated lymphatics in the lymphadenovarix yielded microfilariae indicating the presence of adult gravid female worms in nearby lymphatics. Finally, the diameter of the lymphatic vessels reduced significantly and microfilariae disappeared from the blood following treatment with diethylcarbamazine. The effects of diethylcarbamazine on adult worms has been assessed by the absence of microfilaremia and the filarial dance sign on the scrotal ultrasound [5]. Lymphatic vessel dilation however, is not reported to regress after death of the adult worms [1] but in this patient a dramatic reduction in the size of the lymphatics was observed following treatment.

In conclusion, although scrotal lymphatics are the preferred location for adult *W. bancrofti*, axillary lymphatics may also be affected. The host-parasite interaction in these lymphatics may be different from those in the scrotal lymphatics. This may explain the formation of a lymphadenovarix in the axilla and also the dramatic response to diethylcarbamazine in our patient. The authors recommend an ultrasound guided aspiration of fluid from dilated lymphatics of similar lesions in amicrofilaremic patients from filariasis endemic areas.

## Competing interests

The author(s) declare that they have no competing interests.

## Authors' contributions

AB drafted the manuscript with the help of SCS, SKV and SJ. AB, SCS and SJ conceived and designed the study. AB, SCS and SKV were involved in the acquisition of the data. All authors were involved in analysis and interpretation of the data. All authors read and approved the final manuscript.
